# A high-salt/high fat diet alters circadian locomotor activity and glucocorticoid synthesis in mice

**DOI:** 10.1371/journal.pone.0233386

**Published:** 2020-05-21

**Authors:** Yoko Yokoyama, Takahiro J. Nakamura, Karen Yoshimoto, Honoka Ijyuin, Naoyuki Tachikawa, Haruka Oda, Rena Shiraishi, Kaori Shinohara, Kayo Kumadaki, Shiori Honda, Anna Nakamura, Naho Kitamura, Kazuo Tsubota, Mitsuhiro Watanabe

**Affiliations:** 1 Graduate School of Media and Governance, Keio University, Fujisawa, Kanagawa, Japan; 2 Health Science Laboratory, Keio Research Institute at SFC, Fujisawa, Kanagawa, Japan; 3 Laboratory of Animal Physiology, School of Agriculture, Meiji University, Kawasaki, Kanagawa, Japan; 4 Department of Environment and Information Studies, Keio University, Fujisawa, Kanagawa, Japan; 5 Department of Policy Management, Keio University, Fujisawa, Kanagawa, Japan; 6 Department of Ophthalmology, Keio University School of Medicine, Shinjuku, Tokyo, Japan; University of Lübeck, GERMANY

## Abstract

Salt is an essential nutrient; however, excessive salt intake is a prominent public health concern worldwide. Various physiological functions are associated with circadian rhythms, and disruption of circadian rhythms is a prominent risk factor for cardiovascular diseases, cancer, and immune disease. Certain nutrients are vital regulators of peripheral circadian clocks. However, the role of a high-fat and high-salt (HFS) diet in the regulation of circadian gene expression is unclear. This study aimed to investigate the effect of an HFS diet on rhythms of locomotor activity, caecum glucocorticoid secretion, and clock gene expression in mice. Mice administered an HFS diet displayed reduced locomotor activity under normal light/dark and constant dark conditions in comparison with those administered a normal diet. The diurnal rhythm of caecum glucocorticoid secretion and the expression levels of glucocorticoid-related genes and clock genes in the adrenal gland were disrupted with an HFS diet. These results suggest that an HFS diet alters locomotor activity, disrupts circadian rhythms of glucocorticoid secretion, and downregulates peripheral adrenal gland circadian clock genes.

## 1. Introduction

High salt intake is a prominent lifestyle-related risk factor for hypertension and cardiovascular diseases [1]. A reduction in salt intake at the population level has been considered one of the top five interventions to prevent such non-communicable diseases (NCDs) [2]. To reduce NCDs, the World Health Organization (WHO) intends to reduce salt intake by 30% as one of their nine global targets [3].

Circadian rhythms are observed in various physiological phenomena including blood pressure regulation, cardiovascular physiology, hormone secretion including glucocorticoids and growth hormones, the sleep/wake cycle, thermoregulation, and immune function [4, 5]. Disrupted circadian rhythms are correlated with various diseases including cardiovascular diseases, cancer, and immune disease [4–7]. Circadian rhythms are regulated by a feedback loop, primarily comprising core clock components, BMAL1, CLOCK, CRYs, and PERs [8–13]. BMAL1, CLOCK, NPAS2, and ROR proteins serve as transcriptional activators and PERs, CRYs, and REV-ERB function as inhibitors to produce ∼24-h self-sustained rhythmic transcription of themselves and their target genes [14–17].

A high salt intake is potentially correlated with circadian rhythms. A high-salt diet further enhanced peripheral clock gene expression in mice [18]. A recent forward-genetics-based study reported a role for salt-inducible kinase 3 (SIK3) and Nalcn (Sodium leak channel non-selective protein) in the homeostatic regulation of sleep amount and requirement [19], implying that NaCl levels may be important for regulating circadian rhythms. Other studies reported spontaneously hypertensive rats with advanced circadian clocks in the adrenal gland [20]. Rhythmically secreted glucocorticoids regulate and interact with the body's cell-autonomous clock synchronization [21]. Furthermore, a previous study suggested that administration of steroid hormones altered the rhythms of PER2::LUC expression in peripheral tissue [22]. Since the adrenal grand is an important tissue for orchestrating circadian oscillations [21], it could be an important peripheral tissue for understanding the effects of a high-salt diet on circadian rhythms.

High salt intake is strongly correlated with higher energy intake [23] and a high-fat high-salt diet is suitable for evaluating cardiometabolic diseases [24]. A high-fat diet is one of the nutritional factors affecting circadian rhythms [25, 26]. Previous studies have reported that a high-fat diet ad libitum disrupts feeding-fasting rhythms and dampens daily physiological, metabolic, and gene expression rhythms [26–28]. However, no previous study has examined the effect of a high-fat and high-salt (HFS) diet on peripheral circadian rhythms. Therefore, this study aimed to investigate the effect of an HFS diet on circadian rhythms.

## 2. Materials and methods

### 2.1. Animal studies

**Fig 1:**
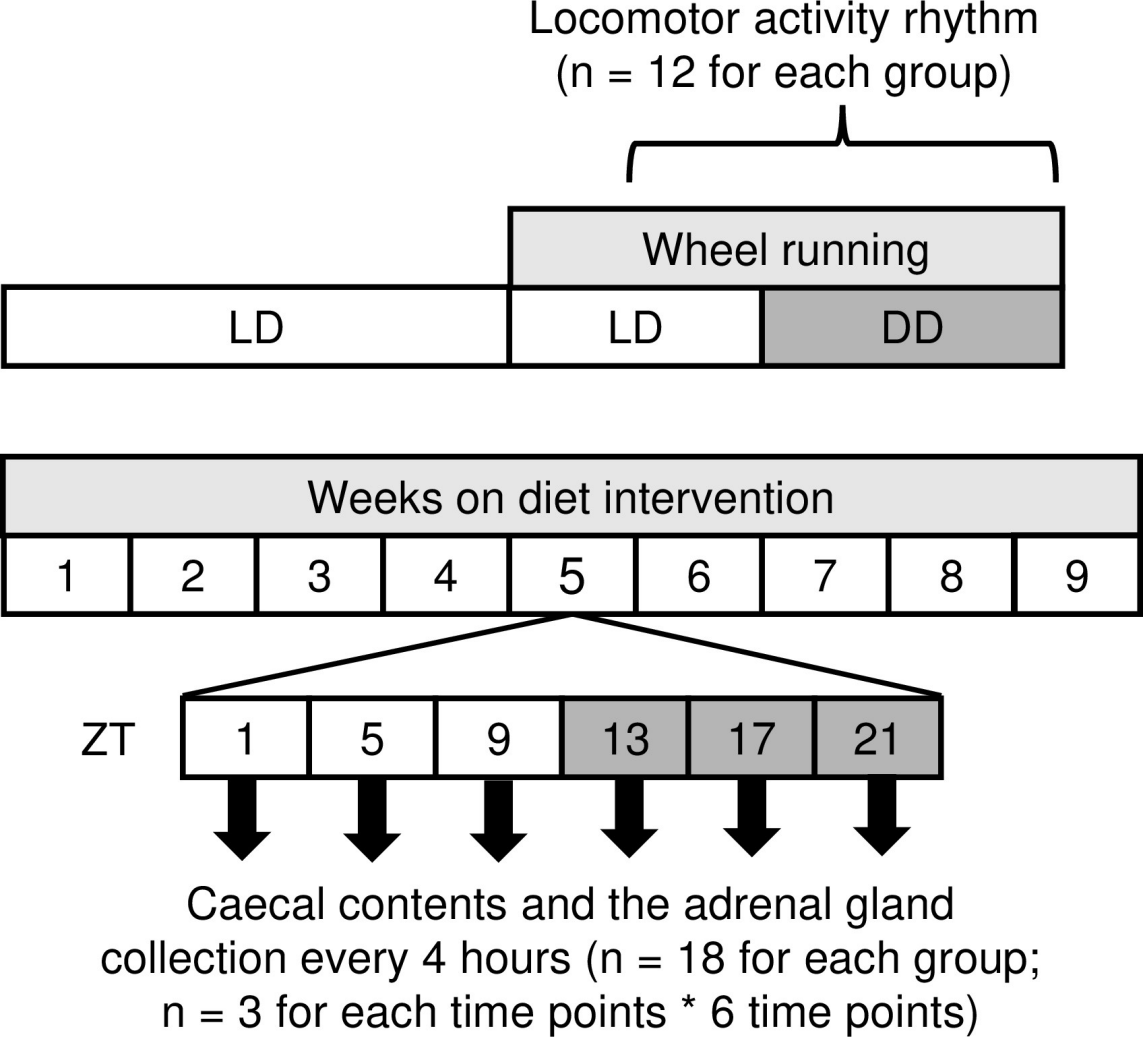
Flow chart of this study.

Male, 6-week-old BALB/cA mice were obtained from CLEA Japan, Inc. (Tokyo, Japan). All mice were housed in a temperature-controlled facility with a 12-h light/dark (LD) cycle and provided ad libitum access to food and water. The control diet and high-fat diet were obtained from Research Diets, Inc. (New Brunswick, NJ, USA). The control diet (D12450B) comprised 20% kcal of protein, 70% kcal of carbohydrate, and 10% kcal of fat. The high-fat diet (D12492) comprised 20% kcal of protein, 20% kcal of carbohydrate, and 60% kcal of fat. For salt treatment, mice were administered diets with 8% (w/w) NaCl compared with 0.3% (w/w) NaCl in the normal diet. Mice were administered a normal salt low-fat diet (C; 0.3% NaCl and 10% kcal fat) or a normal salt high-fat diet (HF; 0.3% NaCl and 60% kcal fat) or an HFS diet (8% NaCl and 60% kcal fat) for four weeks before experiments (Fig 1). All animal experiments were performed according to the institutional guidelines on animal experimentation at Keio University and all animals received humane care. All animal experiments were approved by the Keio University institutional animal care and use committee. All sacrifice was performed under isoflurane anesthesia, and all efforts were made to minimize suffering.

Male BALB/cA mice of 6 weeks of age were fed with a normal salt and control diet (10% kcal fat), a normal salt and high fat diet (60% kcal fat), or a high salt and high fat diet during the experiments. After four weeks of diet intervention, locomotor activity rhythm was assessed through wheel running (n = 12 for each dietary group). Also, mouse caecal contents and the adrenal gland were extracted at Zeitgeber time (ZT) 1, 5, 9, 13, 21 (ZT0 was defined as the time of lights on) (n = 18 for each dietary group; n = 3 for each time points).

### 2.2. Assessment of locomotor activity rhythm

Each mouse (N = 12 for each group) was subjected to a wheel-running activity as previously reported [29]. Each mouse was housed in a separate cage (183 × 340 × 148 mm; CL-0135, CLEA Japan, Tokyo Japan) with a running wheel (12 cm diameter, SANKO, Osaka, Japan). The cages were placed in light-impermeable, ventilated boxes, wherein the light intensity at the bottom of the cage was 200–300 lx. The number of wheel revolutions was determined using a magnet-sensor-activated signal between a button magnet on the running wheel and a magnet relay (59070–010, Littelfuse, Inc., Chicago, IL, USA) fixed on a sidewall of the cage, and was fed into a computer every minute. A chronobiology kit (Stanford Software Systems, Naalehu, HI, USA) and ClockLab software (version 2.72, Actimetrics, Wilmette, IL, USA) were used to curate and visualise the activity data. Periods of wheel-running activity for 15 d under the constant dark (DD) condition were determined using chi-square periodograms. Daily activities and durations of the active phase for 7 d in a normal LD cycle and 15 d in DD were quantified using the activity profile function in ClockLab.

### 2.3. Assessment of glucocorticoid secretion and quantitative RT-PCR (reverse transcription polymerase chain reaction) analysis

Glucocorticoid secretion and qualitative RT-PCR analyses were conducted independent of wheel-running recordings (N = 18 for each dietary group; N = 3 for each ZT). Animals were kept in their LD cycles for four weeks and euthanized at Zeitgeber time (ZT) 1, 5, 9, 13, 17, and 21 (ZT0 was defined as the time of lights on) for the 24-h study. Animals were subjected to isoflurane anaesthesia and caecal contents and the adrenal gland were extracted at ZT 1, 5, 9, 13, 17, and 21 and frozen in liquid nitrogen.

Samples were prepared in accordance with the manufacturer's instructions (https://www.arborassays.com/assets/steroid-solid-extraction-protocol.pdf) [30]. Caecum corticosterone levels were analysed using a corticosterone chemiluminescent immunoassay kit in accordance with the manufacturer's instructions (Arbor Assays, Ann Arbor, MI, USA).

Total RNA was prepared from adrenal grand tissues at ZT 1, 5, 9, 13, 17, and 21. Total RNA was extracted from the tissue samples using the RNeasy Mini Kit (Qiagen, Hilden, Germany). cDNA was synthesized from total RNA with the Prime Script RT Reagent Kit (Takara, Shiga, Japan). For the real-time PCR, gene-specific primers (Table 1) and an SYBR Green Real-Time PCR Master Mix (Takara, Shiga, Japan) were used, and the products were detected using PIKO Real (Thermo Fisher Scientific, Waltham, MA, USA). Relative mRNA expression levels were normalised to that of *Actb* in the same cDNA.

**Table 1:** Primer sequences for RT-PCR analysis.

Gene	Forward primer (5'→3')	Reverse primer (5'→3')
*Actb*	CATCCGTAAAGACCTCTATGCCAAC	ATGGAGCCACCGATCCACA
*Bmal1*	CTCCAGGAGGCAAGAAGATTC	ATAGTCCAGTGGAAGGAATG
*Clock*	GCCTCAGCAGCAACAGCAGC	ACCGCATGCCAACTGAGCGA
*Per1*	AGTTCCTGACCAAGCCTCGTTAG	CCTGCCCTCTGCTTGTCATC
*Per2*	GGGGTGAGATTCGTCATTGAACTTG	AGGACATTGGCACACTGGAAAGAG
*Cry2*	GCTGGAAGCAGCCGAGGAACC	GGGCTTTGCTCACGGAGCGA
*StAR*	AGCCAGCAGGAGAACGGGG	CGCACGCTCACGAAGTCTCG
*Cyp11a*	GAGACACTGAGACTCCACCCCATC	AGACACTGCCGAACACCCCA
*Cyp11b1*	TCACCATGTGCTGAAATCCTTCCA	GGAAGAGAAGAGAGGGCAATGTGT

### 2.4. Statistical analysis

Data are represented as mean ± SEM values. Differences in the locomotor activity, degree of gene expression, caecum corticosterone levels and results of fitted cosine wave were evaluated using the ANOVA and Tukey's post hoc test. Circadian rhythms were statistically analyzed using a fitted cosine wave procedure (Acro program by R.Refinetti: http://www.circadian.org/softwar.html) [31]. A p-value < 0.05 indicated a statistically significant difference. Data analysis was performed using IBM SPSS Statistics Version 25.0 (IBM Corp. Armonk, NY, USA).

## 3. Results

### 3.1. Activity rhythms were not advanced; however, their amplitudes decreased with a HFS diet

**Fig 2:**
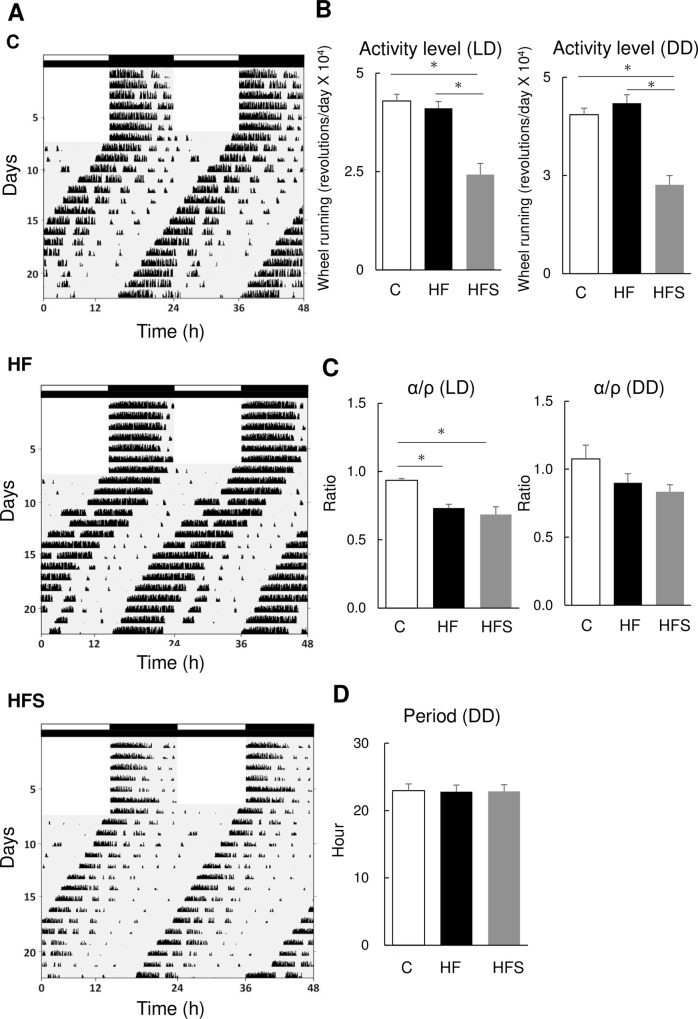
A high-salt high fat diet decreased locomotor activity but did not alter circadian rhythm. (A) Representative actogram in C (Normal Salt and Control diet), HF (Normal salt and high-fat diet), and HFS (high-fat and high-salt diet) groups of mice. (B) Activity levels under LD and DD conditions in different groups. (C) The α/ρ (activity/rest time) ratio under LD and DD conditions among different groups. (D) Circadian period under the DD condition in different groups. The data represent the mean ± standard error of the mean values. *p<0.05.

We examined the effect of the HFS diet on locomotor activity rhythms through wheel-running activity in mice. No differences in locomotor activity patterns were observed among groups during LD and DD conditions, as revealed actogram analysis (Fig 2A), α/ρ ratio at DD (activity time/rest time) (Fig 2C), and period (Fig 2D).

Locomotor activity assessed through wheel running was significantly reduced in HFS mice compared to C or HF fed mice (p < 0.001) (Fig 2A and 2B). This was consistent in both LD and DD conditions. The α/ρ ratio at LD was significantly reduced in HF and HFS fed mice compared with C fed mice.

Average food intake was not different between HF and HFS in LD and DD but it was significantly different between C and HF in LD period (S1A and S1B Fig). In C diet, food intake during LD and DD was significantly different (S1A and S1B Fig) but it was not different in HF and HFS diet.

### 3.2. Rhythms of glucocorticoid levels were disrupted with an HFS diet

**Fig 3:**
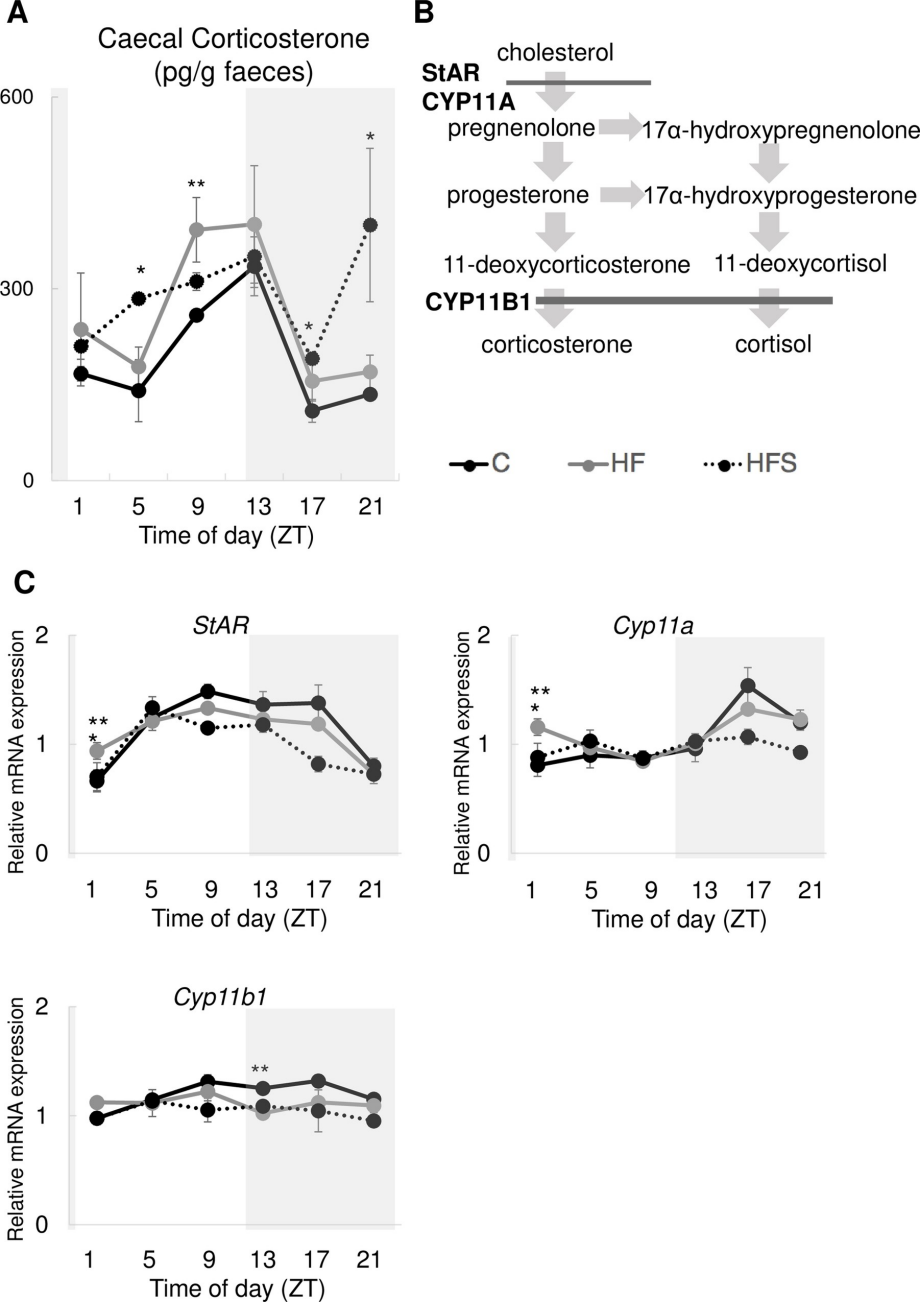
A high-salt high fat diet disrupted the circadian rhythm of caecal corticosterone secretion. (A) diurnal rhythms of caecal corticosterone secretion in different groups. (B) A schematic representation of glucocorticoid synthesis pathways. (C) Relative mRNA expression levels of *StAR*, *Cyp11a*, *Cyp11b1* in the adrenal gland in different groups. Black solid line: C (normal salt and control diet); grey solid line: HF (normal salt and high-fat diet); dotted line: HFS (high-fat and high-salt diet). The data represent the means ± standard error of the mean. *p<0.05 HF vs. HFS, **p<0.05 HF vs. C.

Since glucocorticoid levels are important to orchestrate circadian oscillations, we investigated glucocorticoid rhythms in caecum content (Fig 3A). Caecal glucocorticoid levels displayed nocturnal rhythms, maximum expression at the beginning of the dark period (ZT13) in all groups. However, only the HFS group displayed a bimodal pattern with two peaks per 24-h cycle maximum expression in the beginning of the dark (ZT13) and the end of the dark (ZT21) phase. Low amplitude levels at ZT5 (middle of the light period) (p = 0.023) and ZT17 (middle of the dark period) (p = 0.035) were significantly higher in the HFS group than in the HF group. MESOR (mean statistics of rhythm) of caecal glucocorticoid was significantly lower in C than HF fed mice (p = 0.039) but it was not significantly different between HF and HFS (Table 2).

**Table 2:** 

	Diet	MESOR	Amplitude	Acrophase (h)
Caecal corticosterone	C	190.97 ± 6.84 *	126.01 ± 32.82	10.73 ± 0.93
	HF	255.48 ± 22.03	181.70 ± 33.96	10.47 ± 0.13
	HFS	291.16 ± 19.08	134.19 ± 53.70	12.87 ± 3.65
*StAR* (adrenal gland)	C	1.16 ± 0.04	0.50 ± 0.03 *	11.40 ± 0.83
	HF	1.10 ± 0.04	0.31 ± 0.03	9.93 ± 0.71
	HFS	0.99 ± 0.03	0.35 ± 0.03	8.87 ± 0.13
*Cyp11a* (adrenal gland)	C	1.05 ± 0.02	0.37 ± 0.06	17.53 ± 0.53
	HF	1.09 ± 0.02	0.25 ± 0.01	19.93 ± 0.27
	HFS	0.97 ± 0.03 *	0.21 ± 0.03	16.33 ± 1.75
*Cyp11b1* (adrenal gland)	C	1.19 ± 0.01	0.21 ± 0.02	13.53 ± 1.35
	HF	1.12 ± 0.01	0.14 ± 0.03	7.53 ± 2.53
	HFS	1.04 ± 0.06	0.17 ± 0.04	6.73 ± 3.01

Data shown in Fig 3 analyzed using cosinor analysis. MESOR, mean statistics of rhythm; amplitude, one-half the total peak-through variation; acrophase, hours delay from ZT0; C, control diet; HF, high-fat diet; HFS, high-fat and high-salt diet. Values are means ± SE.

* p < 0.05 (vs. HF).

### 3.3. Expression rhythm of glucocorticoid synthesis-related genes in the mouse adrenal gland was disrupted by an HFS diet

Since the adrenal gland is a key tissue orchestrating circadian oscillations and synthesising corticosteroids (stress hormones), we focused on the adrenal gland. Expression levels of corticosterone synthesis-related genes (Fig 3B) were lower in the HFS group than in the other groups (Fig 3C). *StAR*, a glucocorticoid rate-limiting enzyme, was significantly downregulated in the HFS group rather than the HF group at ZT1 (beginning of the light period) (p = 0.048). Furthermore, first and rate-limiting steroid biosynthesis enzyme, *Cyp11a* were downregulated at ZT1 (p = 0.002) (Fig 3C). MESOR of *Cyp11a* was also significantly lower in HFS fed mice than HF fed mice (p = 0.012) (Table 2). Only HF group significantly upregulated *Cyp11b* than control group (Fig 3C). *StAR* expression levels peaked at 4 h in the HFS group (ZT9 in C and HF compared with ZT5 in HFS diet fed mice) (Fig 3C).

### 3.4. The expression rhythm of clock genes in mouse peripheral tissues was altered with an HFS diet

**Fig 4:**
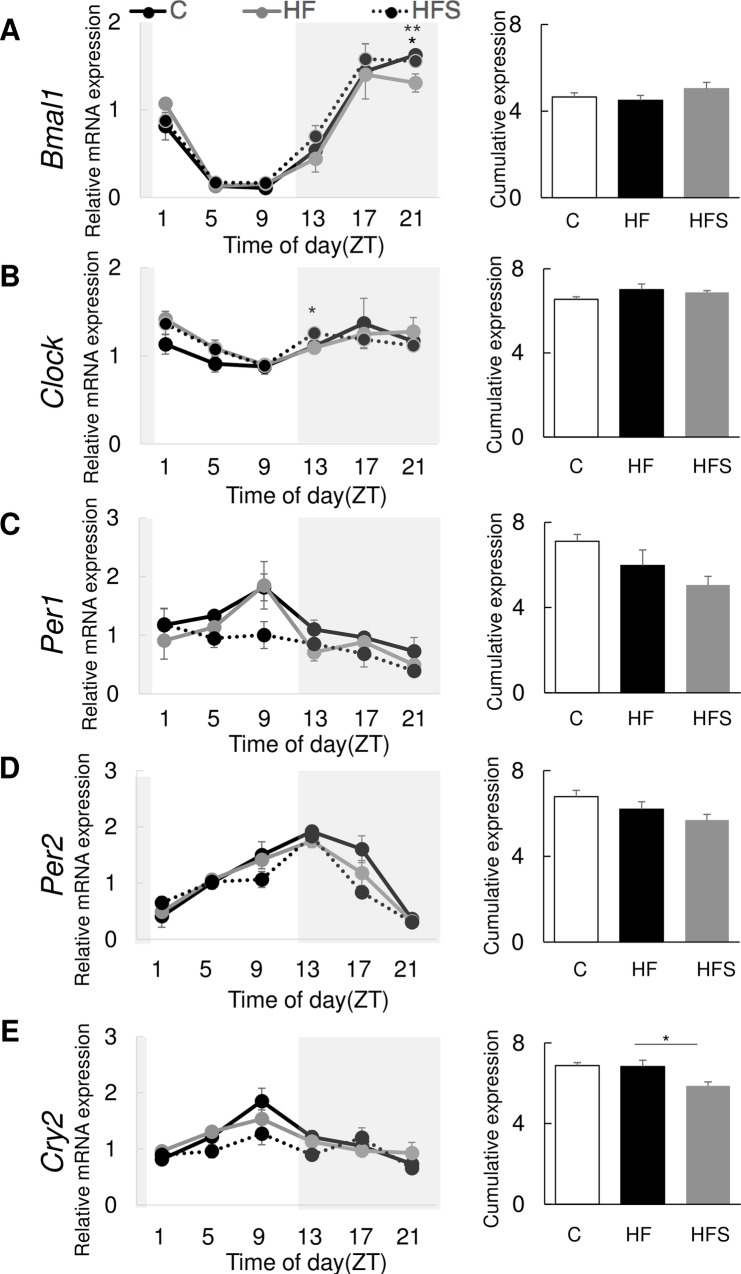
A high-salt and high-fat diet altered peripheral circadian gene expression levels. Relative and cumulative mRNA expression levels in the adrenal gland different groups: (A) *Bmal1*; (B) *Clock*; (C) *Per1*; (D) *Per2*; (E) *Cry2*. Black solid line: C (normal salt and control diet); grey solid line: HF (normal salt and high-fat diet); dotted line: HFS (high-fat and high-salt diet). The data represent the mean ± standard error of the mean values. *p<0.05 HF vs. HFS, **p<0.05 HF vs. C.

We performed a 24-h investigation of the expression of clock genes *Bmal1*, *Clock*, *Per1*, *Per2*, and *Cry2* in the adrenal gland. The HFS diet significantly cumulatively downregulated *Cry2* in the adrenal grand (p = 0.027) (Fig 4E). Also, cosinor analysis found that MESOR of *Cry2* was significantly lower in HFS than HF fed mice (p = 0.027) (Table 3). Furthermore, *Per1* and *Per2* were cumulatively downregulated; however, their expression levels did not significantly differ (Fig 4C and 4D). Expression levels of *Bmal1* in the end of the dark period (ZT21) and *Clock* in the beginning of the dark period (ZT13) differed significantly in the adrenal gland (p = 0.045 and p = 0.022, respectively) (Fig 4A and 4B).

**Table 3:** Cosinor analysis of peripheral circadian genes.

Gene	Diet	MESOR	Amplitude	Acrophase (h)
*Bmal1*	C	0.77 ± 0.03	0.80 ± 0.04	19.53 ± 0.53
	HF	0.75 ± 0.04	0.66 ± 0.01	19.93 ± 0.35
	HFS	0.84 ± 0.05	0.76 ± 0.06	19.13 ± 0.13
*Clock*	C	1.09 ± 0.02	0.33 ± 0.13	12.33 ± 5.68
	HF	1.17 ± 0.05	0.28 ± 0.04	13.93 ± 6.48
	HFS	1.15 ± 0.01	0.26 ± 0.04	20.07 ± 0.93
*Per1*	C	1.18 ± 0.05	0.59 ± 0.23	7.93 ± 0.58
	HF	1.00 ± 0.12	0.70 ± 0.17	7.80 ± 0.46
	HFS	0.84 ± 0.07	0.53 ± 0.08	5.27 ± 1.96
*Per2*	C	1.13 ± 0.05	0.81 ± 0.04	12.33 ± 0.81
	HF	1.03 ± 0.06	0.72 ± 0.05	11.40 ± 0.40
	HFS	0.95 ± 0.04	0.77 ± 0.03	11.13 ± 0.35
*Cry2*	C	1.15 ± 0.02	0.57 ± 0.19	9.80 ± 0.83
	HF	1.14 ± 0.05	0.40 ± 0.03	8.47 ± 0.35
	HFS	0.98 ± 0.03 *	0.39 ± 0.03	11.93 ± 2.24

Data shown in Fig 4 analyzed using cosinor analysis. Details are identical to those in the legend to Table 2. Values are means ± SE.

* p < 0.05 (vs. HF).

## 4. Discussion

This study examined the effects of high-fat and high-salt intake on circadian rhythms in mice. Although no differences in the period of circadian rhythm of locomotor activity were observed, the α/ρ ratio at LD was significantly reduced in HF and HFS fed mice compared with C fed mice. Since it is known that the α/ρ is positively correlated with the circadian period [32, 33], these results suggest that HF and HFS diet may have an effect on the circadian period. However, it is possible that difference of food intake between C and HF may be confounded factors of this result. Furthermore, our results show that an HFS diet reduced wheel-running activity in both LD and DD conditions. In contrast, Oike et al. revealed that high salt administration did not alter locomotor activity including activity levels [18]. This study used mice of the same strain as previously reported [18], but used an HFS diet of 8% w/w salt and 60% kcal fat in this study relative to their study's 4% w/w and 10% kcal, respectively. These differences may account for the differences observed in those studies.

We investigated a potential mechanism explaining lower activity levels in the HFS group than HF group. Firstly, a disrupted association between the central and peripheral clock may have reduced activity levels. The adrenal gland links the central and peripheral clocks through glucocorticoids [34]. However, some circadian rhythms in the expression of clock genes were retained in adrenalectomized mice, and over half of the genes did not show circadian rhythmicity, suggesting that glucocorticoids are one of the links between the central and peripheral clock [35]. Previous studies have reported that high salt intake (4%) for 4 weeks further promotes circadian expression of clock genes in peripheral tissues such as the liver, kidneys, and lungs [36]. Furthermore, our study shows that circadian clock gene expression levels were altered in the adrenal gland. Indeed, a recent study investigated a hypertensive rat model (spontaneously hypertensive rats; SHR) and revealed an abnormal adrenal circadian clock that potentially affects the transcriptional regulation of clock-controlled genes and steroid hormone secretion by the adrenal gland [20].

The adrenal peripheral clock regulates the autonomous circadian rhythm of glucocorticoid secretion by causing rhythmic steroid production [34]. *StAR* is a CLOCK-BMAL1-regulated gene and is a rate limiting enzyme for glucocorticoid synthesis [37, 38]. Although *Bmal1* and *Clock* expression levels were different between the HFS and HF groups in the dark period, a clear association between circadian clock gene expression and *StAR* expression in the adrenal gland was not observed. However, in another first and rate limiting enzyme for glucocorticoid synthesis genes *Cyp11a* [39], significantly lower MESOR was found in HFS than in HF diet fed mice. It is possible that negative feedback of glucocorticoid reduced *Cyp11a* gene expression level in HFS than HF.

Furthermore, adrenal *Cry2* cumulative expression levels decreased in the HFS group. It was confirmed by the result which the MESOR of *Cry2* was significantly lower in HFS than HF fed mice. In mice, the loss of *Cry1* and/or *Cry2* resulted in glucose intolerance and constitutively high levels of circulating corticosterone, suggesting reduced suppression of the hypothalamic-pituitary-adrenal (HPA) axis coupled with increased glucocorticoid transactivation in the liver [40]. *Cry1* and *Cry2* are genetically associated with a glucocorticoid response element (GRE) [40]. *Cry2* expression levels peaked in the HFS group at ZT9 and ZT17, which was advanced by 4 h in comparison with caecal corticosterone peaks. These results suggest that disruption of adrenal circadian *Cry2* expression potentially leads to differences in circulating levels of corticosterone owing to the GRE. Circulating levels of corticosterone may affect locomotor activity levels. A previous study reported that modulation of circadian glucocorticoid oscillation through an enhancement in its amplitude leads to anxiolytic-like behaviour [41]. The present results show that an HFS diet decreases locomotor activity in both light and dark periods with abnormal corticosterone circulation rhythms. Further studies are needed to clarify whether abnormal circulation of corticosterone decrease locomotor activity under normal conditions without anxiety intervention. In addition, it is well known that excessive salt intake affects the renin–angiotensin–aldosterone system (RAAS) [42, 43]. RASS is closely related with HPA and circadian release of glucocorticoids is regulated by HPA, hypothalamic suprachiasmatic nucleus (SCN) signaling, and peripheral adrenal clocks interaction [44]. Previous study shows that hypertensive TGR (mREN-2) 27 (TGR) rats which is a model of upregulated renin-angiotensin system found changes in the clock gene expression in the area postrema [45]. Since RAAS is also located in adrenal gland [46], potential relationship between RAAS and circadian clock are needed to clarify in the future.

Downregulation of clock genes in the HFS group may be associated with reduced locomotor activity. A previous study suggested that circadian gene expression patterns in the mouse central clock predict the levels of locomotor activity [36]. They reported an association between lower circadian gene expression levels and lower locomotor activity [36]. Furthermore, aging is associated with altered locomotor activity rhythms, including decreased amplitudes [47–51]. The HFS diet induced similar effects with aging in terms of decreased amplitudes; however, other changes such as increased fragmentation, shortened or lengthened free-running periods, slower re-entrainment following LD cycle shifts, and altered light sensitivity were not observed. Although we did not examine circadian gene expression patterns in the brain, our data suggest that circadian genes were downregulated in peripheral tissues in the HFS group, wherein locomotor activity levels were lower under both LD and DD conditions. However, in same time, it is possible that the phenotypes of this study in peripheral adrenal clock gene expression may simply be a result of reduced locomotor activity. Further studies are needed to clarify this possible associations.

This study has several strengths. Firstly, this study was conducted under experimental conditions that potentially reduce confounders such as environmental and genetic factors. For instance, temperature and humidity or salt sensitivity would be possibly be those factors. Secondly, we focused on novel outcomes such as the association between circadian rhythms, and high-fat and high-salt intake. The present results suggest that an HFS diet, a prominent public health concern worldwide, potentially affects physical activity levels.

However, this study has the following limitations. Firstly, alterations in feeding pattern that is confounded by the diet and also the switch between LD and DD could influence the overall measures described here, including glucocorticoids and gene expression. Secondly, we analysed the circadian rhythm of glucocorticoid secretion using caecal contents. A previous study compared plasma and faecal corticosterone levels and reported that although the phase was advanced in plasma, followed by faecal corticosterone levels, the circadian rhythm of glucocorticoid secretion was similar in rats [52]. Herein, owing to the high sensitivity of corticosterone to various stressors and the need for its assessment every 4 h, we analysed caecum corticosterone levels; however, the similarity in caecal and faecal corticosterone levels remains unclear in mice. Thirdly, although we investigated locomotor activities under LD and DD conditions, we did not investigate central clock gene expression patterns. Fourthly, this study is based on gene expression analysis in a single inbred strain. Especially, gene expression levels of *StAR* were not well corresponding to the caecum corticosterone levels. It is possible that mRNA levels were not well correlated with protein levels or caecum corticosterone levels were correlated with other factors were unknown. Therefore, the results must be interpreted with caution. Further studies are required to explore the potential mechanisms underlying the association between high-fat and high-salt intake and lower activity levels.

## 5. Conclusions

This study shows that an HFS diet is associated with lower locomotor activity and disrupted circadian rhythms of glucocorticoid synthesis in mice. Further studies are required to clarify the mechanisms underlying these associations and to explore their occurrence in humans.

## Supporting information

S1 Fig Average food intake during LD and DD. (A) Average food intake in C (normal salt and control diet), HF (normal salt and high-fat diet), and HFS (high-fat and high-salt diet) groups of mice during LD. (B) Average food intake during DD. The data represent the mean ± standard error of the mean values. *p<0.05.
(PPTX)
