# Supplementary material for: A high-salt/high fat diet alters circadian locomotor activity and glucocorticoid synthesis in mice
Source: PLoS One. 2020 May 21;15(5):e0233386. doi: 10.1371/journal.pone.0233386 (PMC7241774; doi:10.1371/journal.pone.0233386)
Supplement: S1 Fig — (A) Average food intake in C (normal salt and control diet), HF (normal salt and high-fat diet), and HFS (high-fat and high-salt diet) groups of mice during LD. (B) Average food intake during DD. The data represent the mean ± standard error of the mean values. *p<0.05. (PPTX) [file pone.0233386.s001.pptx]

## Slide 1
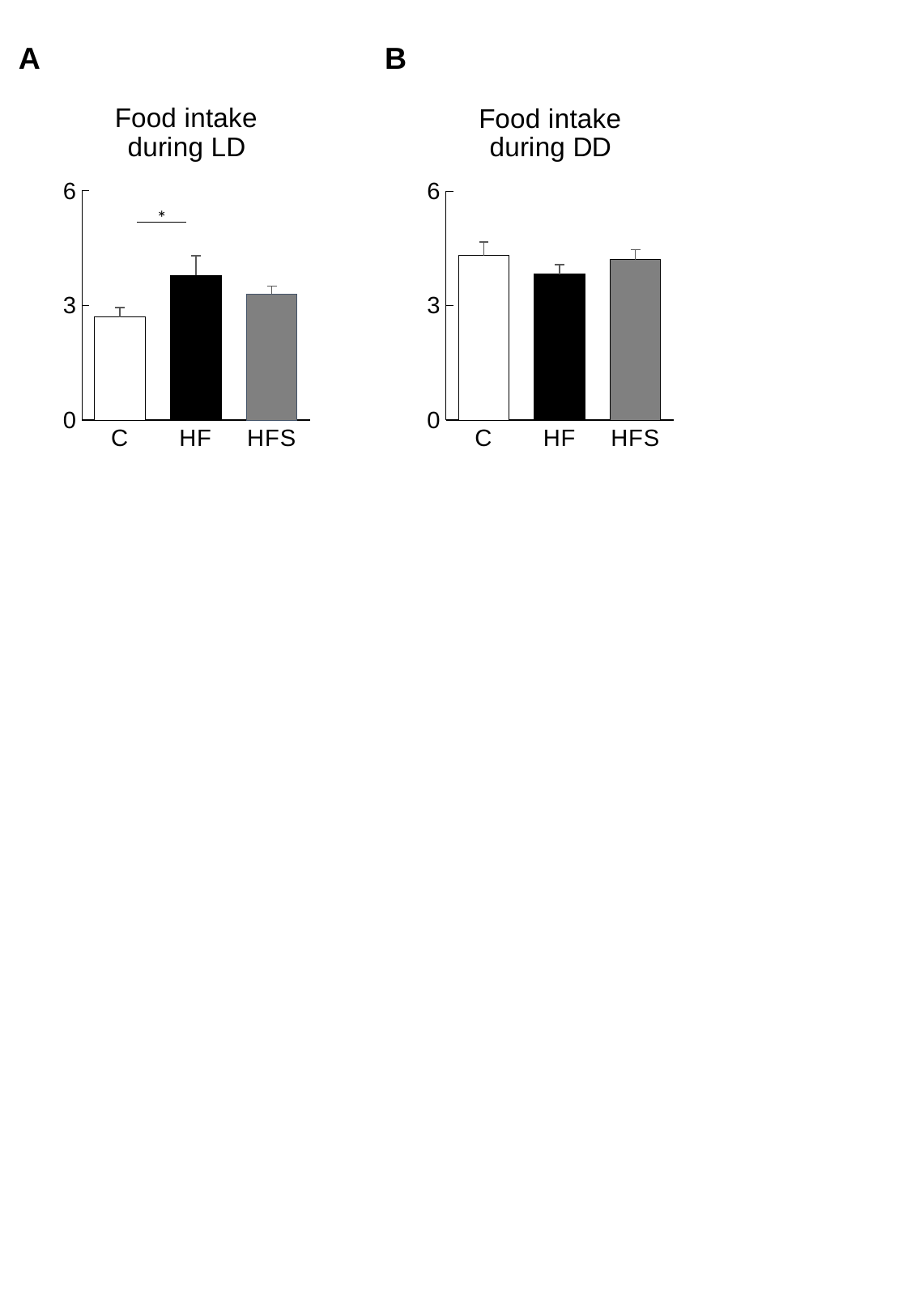

A
B
### Chart: Food intake during LD
| Category | |
|---|---|
| C | 2.697222222222223 |
| HF | 3.7821428571428566 |
| HFS | 3.3037037037037034 |
### Chart: Food intake during DD
| Category | |
|---|---|
| C | 4.314285714285715 |
| HF | 3.8225 |
| HFS | 4.1999999999999975 |*
